# TusA (YhhP) and IscS are required for molybdenum cofactor-dependent base-analog detoxification

**DOI:** 10.1002/mbo3.108

**Published:** 2013-07-29

**Authors:** Stanislav G Kozmin, Elena I Stepchenkova, Roel M Schaaper

**Affiliations:** 1 Laboratory of Molecular Genetics, National Institute of Environmental Health SciencesResearch Triangle Park, North Carolina, 27709; 2 Department of Genetics and Biotechnology, St. Petersburg State University, St. Petersburg 199034, and St. Petersburg Branch of Institute of General Genetics, RASSt. Petersburg 199034, Russia

**Keywords:** 6-*N*-hydroxylaminopurine (HAP) sensitivity, chlorate resistance, IscS cysteine desulfurase, molybdenum cofactor (Moco) biosynthesis, TusA sulfur-carrier protein.

## Abstract

Lack of molybdenum cofactor (Moco) in *Escherichia coli* leads to hypersensitivity to the mutagenic and toxic effects of *N*-hydroxylated base analogs, such as 6-*N*-hydroxylaminopurine (HAP). This phenotype is due to the loss of two Moco-dependent activities, YcbX and YiiM, that are capable of reducing HAP to adenine. Here, we describe two novel HAP-sensitive mutants containing a defect in *iscS* or *tusA* (*yhhP*) gene. IscS is a major L-cysteine desulfurase involved in iron–sulfur cluster synthesis, thiamine synthesis, and tRNA thiomodification. TusA is a small sulfur-carrier protein that interacts with IscS. We show that both IscS and TusA operate within the Moco-dependent pathway. Like other Moco-deficient strains, *tusA* and *iscS* mutants are HAP sensitive and resistant to chlorate under anaerobic conditions. The base-analog sensitivity of *iscS* or *tusA* strains could be suppressed by supplying exogenous L-cysteine or sulfide or by an increase in endogenous sulfur donors (*cysB* constitutive mutant). The data suggest that *iscS* and *tusA* mutants have a defect in the mobilization of sulfur required for active YcbX/YiiM proteins as well as nitrate reductase, presumably due to lack of functional Moco. Overall, our data imply a novel and indispensable role of the IscS/TusA complex in the activity of several molybdoenzymes.

## Introduction

**Figure 1:**
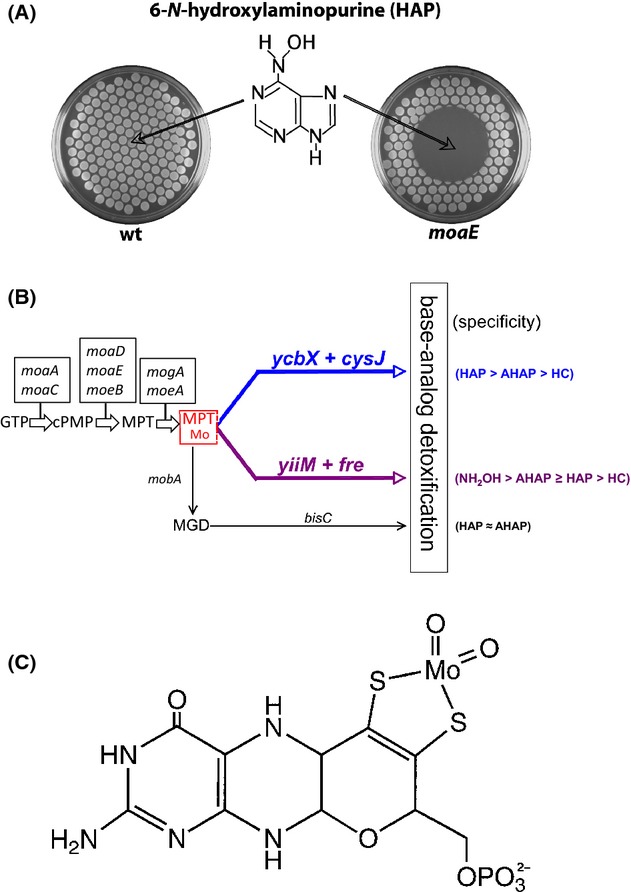
Base-analog sensitivity of *Escherichia coli* deficient in Moco biosynthesis, previously described Moco-dependent pathways for HAP detoxification, and structure of Moco. (A) The chemical structure of HAP and the results of a spot test when HAP (10 μg) was placed in the center of a plate seeded with either the wild-type (wt) strain (NR10836) or its Moco-deficient (Δ*moaE*) derivative (NR16523) using a multiprong replicator device (see Experimental Procedures). (B) Genetic pathways for Moco-dependent base-analog detoxification. The diagram, adapted from Kozmin et al. (2008) and Kozmin et al. (2010), shows some of the synthesis steps of Moco, as well as its use in MPT form, for the YcbX- and YiiM-dependent pathways. The latter two perform in conjunction with flavin reductases CysJ (Kozmin et al. 2010) and Fre (S. G. Kozmin, E. I. Stepchenkova, J. Wang, and R. M. Schaaper, unpublished data) the detoxification (reduction) of 6-*N*-hydroxylaminopurine (HAP), 2-amino-HAP (AHAP), *N*^4^-hydroxycytidine (HC), and hydroxylamine (NH_2_OH). The *bisC* pathway, mediated by biotin sulfoxide reductase, is a minor pathway dependent on the MGD form of Moco, which is only detectable in a *ycbX yiiM* background. The diagram also indicates the differential specificity of the three pathways for the indicated substrates. See text and Discussion for more details. (C) The structure of the Molybdenum cofactor. Shown is a simple Mo-MPT form that may be present in YcbX and YiiM (Kozmin et al. 2008). The Mo atom is coordinated through the dithiolene sulfurs connecting it to the ring system. Different forms of Moco have been described with additional sulfur ligand coordination at the Mo center, as well as nucleotide modifications, like the MGD or MCD dinucleotide form (Schwarz et al. 2009; Iobbi-Nivol and Leimkühler 2012).

Base analogs are modified nucleobases that can substitute for the natural bases in cellular metabolism, and their involvement can lead to toxic and/or mutagenic consequences. Base analogs may be generated in vivo during normal cellular metabolism or by the action of certain chemical and physical factors, such as alkylating agents or ionizing radiation. An important group of mutagenic base analogs are the *N*-hydroxylated derivatives of purines and pyrimidines, such as 6-*N*-hydroxylaminopurine (HAP) (Fig. 1A), 2-amino-HAP (AHAP), and *N*^4^-hydroxycytidine (HC). These agents have been shown to be strong mutagens in bacteria, yeast, and mammalian cells (Barrett 1981; Pavlov et al. 1991; Kozmin et al. 1998; Kozmin et al. 2000). HAP can be produced enzymatically from adenine by hepatic microsomal *N*-hydroxylation (Clement and Kunze 1990) and from the inadvertent use of hydroxylamine in purine biosynthesis (Lieberman 1956). It has also been shown to be a major product of exposure of DNA and nucleobases to peroxyl radicals (Simandan et al. 1998).

Our previous studies have shown that *Escherichia coli* strains lacking molybdenum cofactor (Moco) are hypersensitive to the toxic and mutagenic action of HAP (Fig. 1A) and related *N*-hydroxylated analogs, including AHAP, HC, and hydroxylamine (NH_2_OH) (Kozmin et al. 2000; Kozmin and Schaaper 2007). Subsequently, we demonstrated that Moco-dependent base-analog detoxification is due to action of two Moco-dependent enzymes, YcbX and YiiM, which were shown capable of reducing HAP to nontoxic adenine (Kozmin et al. 2008). Further genetic studies revealed the participation of CysJ flavin reductase as electron donor for the YcbX activity (Kozmin et al. 2010), while Fre flavin reductase is thought to be the likely electron donor for YiiM activity (S. G. Kozmin, E. I. Stepchenkova, J. Wang, and R. M. Schaaper, unpublished data). A third (minor) Moco-dependent HAP-detoxification activity was discovered in a *ycbX, qyiiM*-deficient background, which was identified as the *bisC* gene product (biotin sulfoxide reductase) (Kozmin et al. 2008). The three established pathways for HAP detoxification are outlined in Figure 1B. One important difference between the *ycbX* and *yiiM* pathways and the *bisC* pathway relates to the precise structure of the Moco. Most *E. coli* molybdoenzymes, including BisC, belong to the dimethylsulfoxide (DMSO)-reductase family, which utilize a molybdopterin (MPT)-guanine-dinucleotide (MGD) form as cofactor (Iobbi-Nivol and Leimkühler 2012). While the structure of the cofactor in YcbX and YiiM is not yet established, it is clear that it does not require *mobA*-dependent MGD formation, and it may represent a simple MPT form (Fig. 1B and C) (Kozmin et al. 2008). Consequently, the YcbX and YiiM proteins were suggested to represent members of a distinct novel family of molybdoenzymes (Anantharaman and Aravind 2002; Kozmin et al. 2008). Two mammalian mitochondrial proteins, mARC1 and mARC2, are likely members of the same family (Anantharaman and Aravind 2002; Wahl et al. 2010), as well as a protein (crARC) from *Chlamydomonas reinhardtii* (Chamizo-Ampudia et al. 2011), and all share the ability to reduce *N*-hydroxylated substrates.

As part of a continuing investigation of the cellular factors that determine resistance to toxic *N*-hydroxylated compounds, and to better understand the regulation of Moco and Moco-dependent enzymes, we have undertaken further searches for genetic mutants of *E. coli* that display altered sensitivity to the base-analog HAP. Here, we describe the properties of two such mutants, *tusA* and *iscS*, that express increased sensitivity to HAP and related compounds. While *iscS* encodes a L-cysteine desulfurase involved in various sulfur-dependent activities (Fontecave et al. 2008; Roche et al. 2013), such as iron–sulfur cluster biosynthesis, the only known function for TusA is as a sulfur carrier in the thiomodification of certain tRNAs, where it operates in complex with IscS (Ikeuchi et al. 2006). Our results reveal a novel correlation between the activity of Moco-dependent enzymes and cellular sulfur metabolism.

## Experimental Procedures

### Media and chemicals

Bacteria were cultivated in Luria-Bertani (LB) broth (Miller 1972) or minimal Vogel–Bonner medium (VB) (Vogel and Bonner 1956) containing 0.2% glucose as carbon source and supplemented with 12.5 μg/mL of nicotinamide and 1 μg/mL of thiamine. When indicated, minimal media was also supplemented with 0.33 mmol/L L-cysteine or 2 mmol/L sodium sulfide. Solid media contained 1.5% agar. For selection of antibiotic-resistant clones, media was supplemented with 35 μg/mL of kanamycin or 15 μg/mL of tetracycline, or 100 μg/mL of rifampicin. HAP, in form of free base, was purchased from Midwest Research Institute (Kansas City). All other chemicals were from Sigma-Aldrich.

### Bacterial strains

The *E. coli* strains used in this study are listed in Table 1, along with their source or derivation. All mutagenesis and base-analog sensitivity tests were performed using strain NR10836 and its mutant derivatives. The *tusA*::*kan* mutant was obtained from a genome-wide search for HAP-sensitive mutants using the EZ-Tn5™ <R6Kγori/KAN-2>Tnp Transposome™ Kit from Epicentre, Madison, WI. The Δ*iscS*, Δ*iscU*, Δ*iscA,* Δ*iscUA,* Δ*sufS,* Δ*sufA,* Δ*csdA(b2810),* Δ*nfuA,* Δ*tusBCD,* and Δ*tusE* deletions were generated in strain BW25113/pKD46 by the polymerase chain reaction (PCR)-based gene replacement method of (Datsenko and Wanner 2000), using either the Kan^r^ module of plasmid pKD13 (Datsenko and Wanner 2000) or the *tetA tetR* tetracycline-resistant (Tet^r^) module of transposon Tn*10* as a template. Primers for the PCR reactions were (upper case letters indicate the sequences of Tn*10* or pKD13) *iscS*-p1 (5’-gta agc cat tat aaa ttc tcc tga ttc cga tac cga tta atg atg agc ccG TGT AGG CTG GAG CTG CTT CG-3’) and *iscS*-p4 (5’-ggt agc ctg att cct tgc att gag tga tgt acg gag ttt ata gag caa tgA TTC CGG GGA TCC GTC GAC C-3’) for generating Δ*iscS*::*kan*; *iscU*-p1 (5’-caa ctc tta ttt tgc ttc acg ttt gct ttt ata gtc cgc aat ggc ggc ttG TGT AGG CTG GAG CTG CTT CG-3’) and *iscU*-p4 (5’-cgg aat cag gag aat tta taa tgg ctt aca gcg aaa aag tta tcg acc atA TTC CGG GGA TCC GTC GAC C-3’) for Δ*iscU*::*kan*; *iscA*-p1 (5’-aaa ccc cac gcg cag gcg acc acg gtg ggg tta tcg gta tgc gca tca aaG TGT AGG CTG GAG CTG CTT CG-3’) and *iscA*-p4 (5’-gga cta taa aag caa acg tga agc aaa ata aga gtt gag gtt tgg tta tgA TTC CGG GGA TCC GTC GAC C-3’) for Δ*iscA*::*kan*; *iscU*-p1 and *iscA*-p4 for Δ*iscUA*::*kan*; *sufS*-p1 (5’-gac aca tcg cag gga cgt tgt aat agg cca tca atg gca ttg cgc agt gaG TGT AGG CTG GAG CTG CTT CG-3’) and *sufS*-p4 (5’-gca aga tga ttt ttt ccg tcg aca aag tgc ggg ccg act ttc cgg tgc ttA TTC CGG GGA TCC GTC GAC C-3’) for Δ*sufS*::*kan*; *sufA*-p1 (5’-ttt tga cat cgt cag ttg ctt cag tat tac gag aca tag tac cgc cta taG TGT AGG CTG GAG CTG CTT CG-3’) and *sufA*-p4 (5’-cac taa cat gct gtt ata cgc tga aag cga tga agt gag gta aat cga tgA TTC CGG GGA TCC GTC GAC C-3’) for Δ*sufA*::*kan*; *csdA*-p1 (5’-aag ccg agg agt acc atg aac gtt ttt aat ccc gcg cag ttt cgc gcc caG TGT AGG CTG GAG CTG CTT CG-3’) and *csdA*-p4 (5’-taa tcc acc aat aat tcc agc gcg cgg tca acg gca ttc acc agc gca tcA TTC CGG GGA TCC GTC GAC C-3’) for Δ*csdA*::*kan*; *nfuA*-p1T (5’-ggc gta tta taa cca act aaa ata gtc aac tat tag gcc att act atg atA AGA GGG TCA TTA TAT TTC G-3’) and *nfuA*-p4T (5’-tta gcg tcg cat cgg gca atc tac aaa aga ggg gat aac tta gta gta ggA CTC GAC ATC TTG GTT ACC G-3’) for Δ*nfuA*::*tet*; *tusBCD*-p1T (5’-ttt agg gcg atg ccg aaa agg tgt caa gaa ata tac aac gat ccc gcc ata AGA GGG TCA TTA TAT TTC G-3’) and *tusBCD*-p4T (5’-ctc tac atc cgc cag ttc aag agc ggt gat ttc cag ggg caa gat aag taA CTC GAC ATC TTG GTT ACC G-3’) for Δ*tusBCD*::*tet*; and *tusE*-p1 (5’-cct cgg ggg aat taa ccg att ttc gta tcc gtt aaa tac att tta ccg gtG TGT AGG CTG GAG CTG CTT CG-3’) and *tusE*-p4 (5’-tgt tac act gcc gcc agt ctg att aag gta gta ata att atg ctg atc ttA TTC CGG GGA TCC GTC GAC C-3’) for Δ*tusE*::*kan*. The resulting deletion–insertions were then transferred into strain NR10836 by P1 transduction using P1*virA,* and the Kan^r^ modules, if necessary, were eliminated using pCP20 plasmid, as described by Datsenko and Wanner (2000) (see Table 1).

**Table 1:** *Escherichia coli* strains used in this study

Strain	Genotype	Reference or derivation
BW25113 [pKD46]	*lacI*^q^ *rrnB*_T14_ Δ*lacZ*_WJ16_ *hsdR514* Δ*araBAD*_AH33_ Δ*rhaBAD*_LD78_ [pKD46]	(Datsenko and Wanner 2000)
NR10836	*ara thi* Δ(*pro-lac*) F'CC106	(Kozmin et al. 2000)
NR15995	NR10836, but Δ*moeB*::*kan*	(Kozmin et al. 2000)
NR15996	NR10836, but Δ*moaE*::*kan*	(Kozmin and Schaaper 2007)
NR16042	NR10836, but Δ*sufS*::*kan*	This study
NR16045	NR10836, but Δ*csdA*	This study
NR16046	NR10836, but Δ*sufS*	This study
NR16072	NR10836, but ΔiscU*	This study
NR16075	NR10836, but Δ*csdA* Δ*sufS*::*kan*	NR16045 × P1/NR16042
NR16195	NR10836, but *cysB*^c^(T149P) *zcj-233*::Tn*10*	(Kozmin et al. 2010)
NR16263	NR10836, but ΔiscS*	This study
NR16523	NR10836, but Δ*moaE*	NR15996 but kanamycin sensitive**
NR16539	NR10836, but ΔiscS* *cysB*^c^(T149P) *zcj-233*::Tn*10*	NR16263 × P1/NR16195 and selection on tetracycline and 1,2,4-triazole (Kozmin et al. 2010)
NR16758	NR10836, but ΔiscS* Δ*moaE*::*kan*	NR16263 × P1/NR15996
NR17603	NR10836, but *tusA*::*kan*	This study
NR17604	NR10836, but ΔiscS* *tusA*::*kan*	NR16263 × P1/NR17603
NR17605	NR10836, but Δ*moaE tusA*::*kan*	NR16523 × P1/NR17603
NR17606	NR10836, but ΔiscS* Δ*moaE*	NR16758 but kanamycin sensitive**
NR17607	NR10836, but ΔiscS* Δ*moaE tusA*::*kan*	NR17606 × P1/NR17603
NR17613	NR10836, but Δ*iscA*::*kan*	This study
NR17614	NR10836, but Δ*tusE*::*kan*	This study
NR17615	NR10836, but Δ*sufA*::*kan*	This study
NR17616	NR10836, but Δ*nfuA*::*tet*	This study
NR17617	NR10836, but Δ*tusBCD*::*tet*	This study
NR17618	NR10836, but ΔiscA*	NR17613 but kanamycin sensitive**
NR17619	NR10836, but Δ*tusE*::*kan* Δ*tusBCD*::*tet*	NR17614 × P1/NR17617
NR17632	NR10836, but Δ*iscUA*::*kan*	This study
NR17633	NR10836, but Δ*iscUA*::*kan* Δ*nfuA*::*tet*	NR17632 × P1/NR17616
NR17634	NR10836, but ΔiscA* Δ*nfuA*::*tet*	NR17618 × P1/NR17616
NR17635	NR10836, but Δ*sufA*::*kan* Δ*nfuA*::*tet*	NR17615 × P1/NR17616

* In-frame deletions.

** Elimination of the *kan*^r^ module from *moaE* using plasmid pCP20 as described by Datsenko and Wanner (2000).

### Spot test for HAP sensitivity

Stationary *E. coli* cultures grown in LB were diluted in 0.9% NaCl to an OD_600_ = 0.1 and transferred to VB plates using a multiprong replicator device (∼0.1 mL total per plate). After the spots had dried, a few microliters of a 1-mg/mL solution of HAP in DMSO was spotted onto the center of the plate. The plates were incubated overnight at 37°C and inspected the next day for zones of inhibition.

### Test for chlorate sensitivity

Approximately 10^3^ cells were plated on LB plates containing 0.2% KClO_3_ (Miller 1972). The plates were incubated under anaerobic conditions using a Becton Dickinson (Franklin Lakes, NJ) BBL gas pack anaerobic system for 12 h, after which they were incubated aerobically for an additional 6–10 h. Under these conditions, chlorate-sensitive strains do not form colonies, whereas chlorate-resistant strains plate with essentially 100% efficiency.

### Mutant frequency determinations

For each strain, six independent 1-mL VB cultures supplemented (or not) with 0.75 ng/mL of HAP were started from ∼10^5^ cells. The cultures were grown with shaking for 48 h at 37°C. Mutant frequencies were determined by plating of 10–200 μL (depending on strain and presence of the mutagen in the growth media) on an LB-rifampicin plate (to obtain the number of rifampicin-resistant cells per culture) and of 50 μL of a 10^−6^ dilution on LB plates (to obtain the total number of cells per culture).

## Results

### TusA and iscS mutants are base-analog hypersensitive

**Figure 2:**
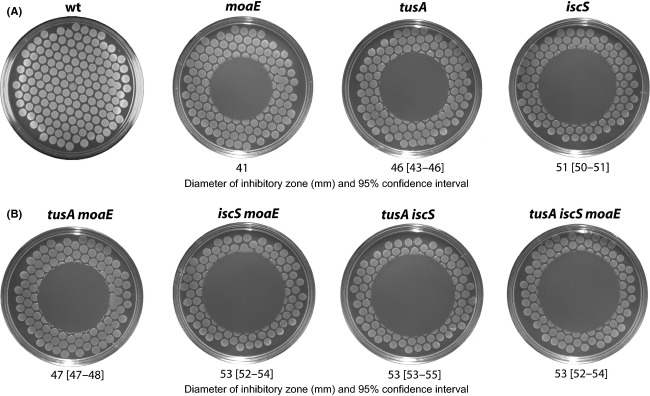
Sensitivity of wild-type *Escherichia coli* and isogenic derivatives to the toxic effects of HAP. (A) We show the wild-type (wt) strain (NR10836), and the single Δ*moaE* (NR16523), *tusA*::*kan* (NR17603), and Δ*iscS* (NR16263) mutants. (B) We present the double *tusA*::*kan* Δ*moaE* (NR17605), Δ*iscS* Δ*moaE*::*kan* (NR16758), *tusA*::*kan* Δ*iscS* (NR17604) strains, and the triple *tusA*::*kan* Δ*iscS* Δ*moaE* mutant (NR17607). Cell suspensions were transferred using a multiprong replicator to VB plates, and 5 μg of HAP (in DMSO solution) was spotted onto the center of each plate. The plates were incubated overnight at 37°C and inspected the next day for zones of inhibition. For each strain, a median value of the inhibitory zone and a nonparametric 95% confidence interval (Altman 1991) obtained from 10 to 20 experiments are noted below the plate. In the case of *moaE* strain, the confidence interval margins equaled the median value.

A genome-wide search for HAP-sensitive mutants using random transposon insertion mutagenesis yielded a mutant carrying a defect in the *tusA* (formerly *yhhP*) open reading frame. This mutant was hypersensitive to HAP (Fig. 2A) as well as to the related *N*-hydroxylated analogs AHAP, HC, and hydroxylamine (data not shown), as previously established for Moco-deficient strains like the *moaE* strain in Figure 1A. (Kozmin et al. 2000, 2008; Kozmin and Schaaper 2007). In fact, the *tusA* strain appeared slightly more sensitive than the *moaE* strain, based on the diameter of the zone of inhibition (46 mm vs. 41 mm) (Fig. 2A). Part of this increased sensitivity of the *tusA* strain may result from a slight growth defect of this strain also reported by others (Yamashino et al. 1998; Ishii et al. 2000). *TusA* encodes an 81-amino acid polypeptide that physically interacts with IscS. The latter is an L-cysteine desulfurase that is a key enzyme in the biosynthesis in iron–sulfur clusters and some other sulfur-related activities (Fontecave et al. 2008; Roche et al. 2013). In particular, the TusA-IscS complex was found to play an essential role in a tRNA uridine thiomodification pathway, in which TusA functions as a sulfur-carrying protein (Ikeuchi et al. 2006).

To investigate the possible role of IscS in base-analog detoxification, we also deleted this gene. As *iscS* is the second gene in an operon containing up to eight genes (Zheng et al. 1998) a precise in-frame *iscS* deletion was created (see Experimental Procedures). We found that the *iscS* mutant, like *tusA*, conferred HAP hypersensitivity (Fig. 2A) as well as sensitivity to AHAP, HC, and hydroxylamine (data not shown). Like for the *tusA* mutant, the *iscS* mutant displayed a reduced growth rate, which may likewise account for the increased apparent HAP sensitivity relative to the Moco-deficient *moaE* mutant (51 mm vs. 41 mm).

### iscS and tusA operate within the Moco pathway

Previous studies on HAP sensitivity defined two parallel pathways for HAP detoxification, dependent on the YcbX and YiiM proteins (Fig. 1B), as well as one minor pathway depending on the BisC protein (Kozmin et al. 2008), each of which was dependent on the availability of the Moco factor. Thus, it was of interest to determine whether the role of the TusA and IscS proteins is related to these pathways. In Figure 2, we show the HAP sensitivity of the *tusA* or *iscS* defects alone or in combination with the *moaE* defect. The *tusA moaE* double mutant showed essentially equal sensitivity to the single *tusA* mutant (47 mm vs. 46 mm). Likewise, the *iscS moaE* double mutant displayed similar activity as the single *iscS* mutant (53 mm vs. 51 mm). These results suggest that the *tusA* and *iscS* defects are likely epistatic to the *moaE* defect. This is further confirmed by the identical sensitivity of the triple *tusA iscS moaE* mutant relative to the double *tusA iscS* mutant (Fig. 2). Although the *iscS* and *tusA* strains appeared more HAP sensitive than the single *moaE* mutant, this may be due to the slower growth observed for *iscS* and *tusA* mutants, which may amplify their sensitivity in this test.

**Figure 3:**
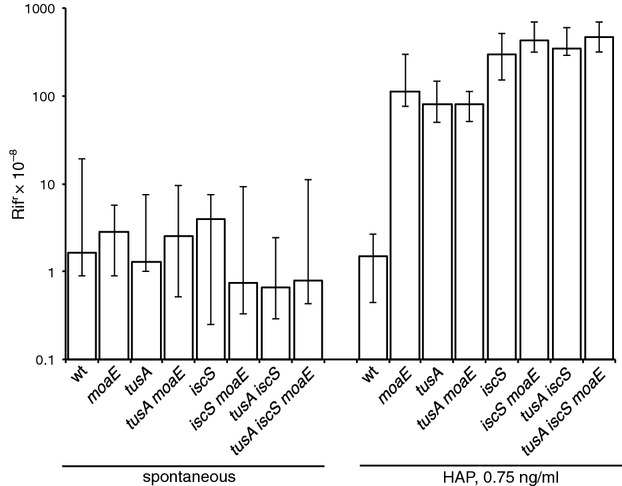
Spontaneous and HAP-induced mutability of *Escherichia coli* strains (frequency of rifampicin-resistant mutants). Strains used are wild type (wt) (NR10836), Δ*moaE* (NR16523), *tusA*::*kan* (NR17603), *tusA*::*kan* Δ*moaE* (NR17605), Δ*iscS* (NR16263), Δ*iscS* Δ*moaE*::*kan* (NR16758), *tusA*::*kan* Δ*iscS* (NR17604), and *tusA*::*kan* Δ*iscS* Δ*moaE* (NR17607). Approximately 10^5^ cells of each strain were inoculated into VB media without or with 0.75 ng/mL of HAP. The cultures were incubated 48 h at 37°C with agitation. Mutant frequencies were determined by plating appropriate culture volumes on LB-rifampicin plates (to obtain the number of Rif^r^ cells per culture) and on LB plates (to obtain the total number of cells per culture). Plotted are the median frequencies of Rif^r^ mutants obtained in six independent cultures as well as the nonparametric 95% confidence intervals (Altman 1991).

The epistasis of the various pathways was also investigated by looking at the mutagenic response of the various strains upon exposure to HAP. The results shown in Figure 3 indicate that the *tusA moaE* combination was equally susceptible to HAP as the single *tusA* (or *moaE*) mutant, and that the *iscS moaE* double mutant, as well as the *iscS tusA moaE* triple mutant, was equally susceptible as the single *iscS* or the double *iscS tusA* mutant (Fig. 3). These data likewise support the operation of TusA and IscS within the Moco pathway. As in the toxicity test, *iscS* strains displayed slightly greater mutability than the *moaE* (and also *tusA*) strain, likely reflecting some indirect sensitization due to the pleiotropic characteristics of *iscS* (Fontecave et al. 2008; Roche et al. 2013).

### Chlorate resistance of tusA and iscS strains

**Figure 4:**
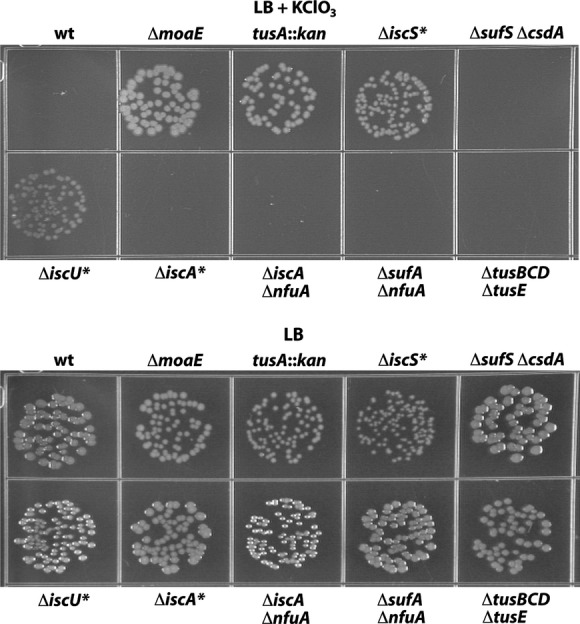
Chlorate sensitivity of *Escherichia coli* strains. A suspension of each tested strain (50–100 cells in 10 μL) was placed on an LB plate or an LB plate containing 0.2% potassium chlorate (KClO_3_). The plates were incubated anaerobically at 37°C. The strains used were wt (NR10836), Δ*moaE* (NR16523), *tusA* (NR17603)*,* Δ*iscS* (NR16263)*,* Δ*sufS* Δ*csdA* (NR16075), Δ*iscU* (NR16072)*,* Δ*iscA* (NR17618)*,* Δ*iscA* Δ*nfuA* (NR17634), Δ*sufA* Δ*nfuA* (NR17635), and Δ*tusBCD* Δ*tusE* (NR17619). See text for further details. Genes indicated with asterisks indicate “in-frame” deletions (see Experimental Procedures).

One major hallmark of lack of Moco is resistance to chlorate under anaerobic conditions (Shanmugam et al. 1992). Sensitivity to chlorate is generally attributed to the activity of nitrate reductase, Moco-dependent activities reducing chlorate to toxic chlorite (Stewart and MacGregor 1982). We tested the *tusA* and *iscS* mutants whether they are, like the previously established Moco-deficient strains, also resistant to chlorate. As shown in Figure 4 (top panel), the *tusA* and *iscS* strain are chlorate resistant, like the control Δ*moaE* strain, indicating that they are also deficient in nitrate reductase activity.

### Inorganic sulfide or L-cysteine partially suppress the HAP sensitivity of iscS or tusA mutants

**Figure 5:**
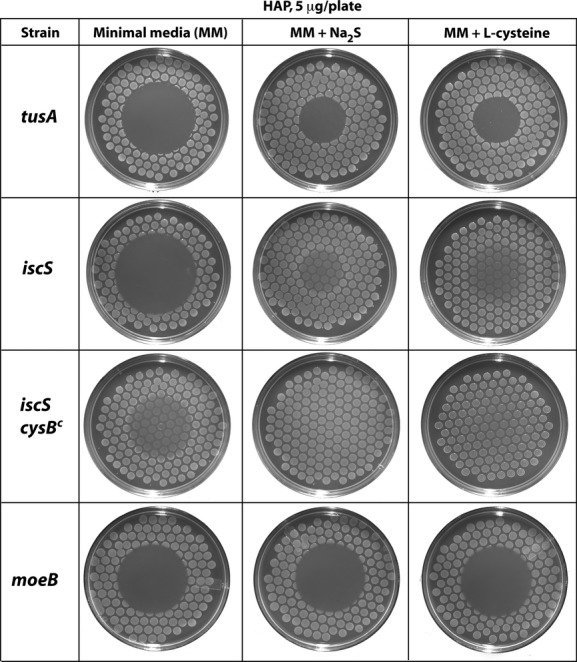
The effect of sulfide and L-cysteine on HAP sensitivity of *tusA* and *iscS* strains. The strains used were *tusA* (NR17603), Δ*iscS* (NR16263), Δ*iscS cysB*^c^ (NR16539), and Δ*moeB* (NR15995). Cell suspensions were transferred to VB plates with or without 2 mmol/L sodium sulfide or 0.33 mmol/L L-cysteine and 5 μg of HAP (in DMSO solution) was spotted onto the center of each plate (see Experimental Procedures for details).

A unique phenotype that we discovered associated with the *iscS* or *tusA* deficiencies is that their HAP sensitivity could be suppressed by addition of sulfide or L-cysteine to the medium (see Fig. 5). This phenotype is not observed for other HAP-hypersensitive mutants, like *moa*, *moe,* or *ycbX* (see *moeB* in Fig. 5). The HAP sensitivity of *iscS* or *tusA* strains is also partially suppressed in strains carrying the constitutive *cysB*^c^ (T149P) allele (Kozmin et al. 2010). In this strain lack of repression of L-cysteine biosynthesis leads to overproduction of L-cysteine (and, likely, sulfide) (Kredich 1996). These observations suggest a defect in *iscS* and *tusA* strains in the mobilization of sulfur required for the activity of the YcbX/YiiM proteins. This function may parallel the described function of the TusA-IscS complex in the thiolation of certain unique tRNAs (Ikeuchi et al. 2006).

### No requirement for alternative L-cysteine desulfurases CsdA and SufS

**Figure 6:**
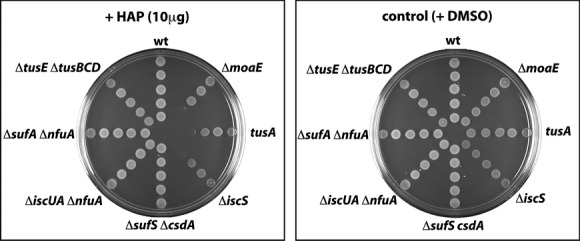
HAP sensitivity of *Escherichia coli* strains carrying defects in the alternative SufS and CsdA cysteine desulfurases, defects in the TusBCDE components of the tRNA thiomodification system, or defects in the IscU, IscA, SufA, and NfuA components of iron–sulfur cluster biogenesis systems. Strains used are wt (NR10836), Δ*moaE* (NR16523), Δ*iscS* (NR16263), Δ*sufS* Δ*csdA* (NR16075), *tusA* (NR17603)*,* Δ*tusBCD* Δ*tusE* (NR17619), Δ*iscUA* Δ*nfuA* (NR17633), and Δ*sufA* Δ*nfuA* (NR17635). A cell suspension of each strain was applied in a series of spots radiating out from the center of the plate. Ten micrograms of HAP (in DMSO solution) or DMSO alone was spotted onto the center of the plate.

Other than IscS, *E. coli* possesses two additional L-cysteine desulfurases, CsdA and SufS (Fontecave et al. 2008; Roche et al. 2013). While IscS alone appears to be fully sufficient for HAP resistance, it seemed worthwhile to check the effect of the additional cysteine desulfurases. Our experiments showed that the single *csdA* and *sufS* mutants as well as the *csdA sufS* double mutant were fully HAP resistant (Fig. 6) as well as chlorate sensitive (Fig. 4), like a wild-type strain. Thus, the postulated need for sulfur transfer in the Moco-dependent activities is fully served by the IscS cysteine desulfurase.

### Role of iron–sulfur cluster biosynthesis in base-analog resistance

IscS is a major L-cysteine desulfurase involved in iron–sulfur cluster biosynthesis (Fontecave et al. 2008; Roche et al. 2013). The first step of Moco biosynthesis requires MoaA protein (Fig. 1B), which contains a catalytically essential iron–sulfur cluster (Schwarz et al. 2009; Iobbi-Nivol and Leimkühler 2012). Important iron–sulfur clusters are also present in nitrate reductases (Schwarz et al. 2009; Iobbi-Nivol and Leimkühler 2012) as well as YcbX protein (Kozmin et al. 2008; J. Wang and R. M. Schaaper, unpublished data). Thus, the observed sensitivity of the Δ*iscS* strain to HAP, as well as its chlorate-resistant phenotype, may be due to a defect in the iron–sulfur cluster of one or more of these enzymes. During cluster biosynthesis, L-cysteine desulfurase provides a sulfur atom to a “scaffold” protein enabling synthesis of a nascent cluster (Fontecave et al. 2008; Roche et al. 2013). The cluster is then transferred to the target apoprotein, either directly or perhaps mediated through one of several carrier proteins. Several possible scaffold and carrier proteins have been described in *E. coli*, including IscU, IscA, SufA, and NfuA (Fontecave et al. 2008; Roche et al. 2013). To check their role, we inactivated the corresponding genes. No increase in HAP sensitivity was observed for any of these mutants, including several of their double mutants (Fig. 6). The strains were also chlorate sensitive (Fig. 4), with the exception of *iscU*. The *iscU* mutant displays a “split” phenotype: IscU is not required for HAP resistance, but is required, at least partially, for nitrate reductase activity (note the reduced size of the chlorate-resistant colonies in Fig. 4). Despite this split phenotype (see also Discussion), the overall results are strongly supportive of the idea that lack of iron–sulfur cluster biosynthesis per se is not the main reason for the HAP sensitivity of *iscS* and *tusA* mutants.

### The effect of TusA is independent of the TusBCD-TusE sulfur transfer complex

The main role that is currently described for TusA is to act as sulfur-carrying protein in the 2-thiolation of 5-methylaminomethyl-2-thiouridylate (mnm^5^s^2^U) at the wobble position of certain tRNAs (Ikeuchi et al. 2006). In this pathway, TusA first accepts sulfur from IscS, which is then transferred via TusBCDE proteins to mnm^5^s^2^U methyltransferase MnmA, which ultimately performs the tRNA thiomodification (Ikeuchi et al. 2006). In our study, inactivation of *tusBCD* and *tusE* produced neither HAP sensitivity (Fig. 6) nor chlorate resistance (Fig. 4). This indicates that, in contrast to the tRNA thiomodification process, the activity of TusA in the Moco pathway does not require the TusBCDE proteins.

## Discussion

In our efforts to understand the mechanisms by which cells can detoxify mutagenic and toxic chemicals such as *N*-hydroxylated compounds we have previously characterized novel activities that depended on at least two proteins, YcbX and YiiM, both requiring the Moco. Mammalian enzymes, termed mARC (Havemeyer et al. 2006), and a green algal protein, crARC (Chamizo-Ampudia et al. 2011) have been described that may be similarly involved in a variety of detoxification reactions, specifically *via* reduction of *N*-hydroxylated substrates to the corresponding amines (Havemeyer et al. 2006, 2010; Gruenewald et al. 2008; Wahl et al. 2010; Chamizo-Ampudia et al. 2011; Kotthaus et al. 2011; Krompholz et al. 2012; Neve et al. 2012). The genetic approaches that we have followed in the bacterium *E. coli* have proven useful in identifying (a) the involvement of Moco as cofactor (Kozmin et al. 2000; Kozmin and Schaaper 2007), (b) the identity of the responsible Moco-dependent enzymes YcbX, YiiM, and BisC (Kozmin et al. 2008), and (c) additional factors involved in these reactions, such as CysJ protein, which functions as a specific electron donor for the YcbX-mediated reduction reaction (Kozmin et al. 2010). In the present study, we have identified two additional factors relevant to these processes, namely the TusA and IscS proteins, which are known to be involved in sulfur mobilization. We have shown that these two proteins act within the previously defined Moco-dependent pathways and, as will be argued below, within the biosynthesis pathway for Moco, most likely in the introduction of the dithiolene sulfur atoms of the MoCo molecule. This functioning of IscS and TusA has also been described recently by Dahl et al. (2013) using more biochemical approaches.

Biosynthesis of Moco is an area of active research (Schwarz et al. 2009; Iobbi-Nivol and Leimkühler 2012). Moco synthesis starts with guanosine-5'-triphosphate (GTP), which is converted to an intermediate called cPMP (cyclic pyranopterin monophosphate) by MoaA and MoaC proteins (Fig. 1B); in the next step the MoaDE/MoeB proteins convert cPMP to the MPT by addition of the dithiolene sulfurs (Fig. 1C), which are needed for coordinating the Molybdenum atom (Moco). Various forms of Moco exist depending on further ligand coordination at the Mo center, as well as possible formation of a dinucleotide with guanosine monophosphate (GMP) or cytidine monophosphate (CMP), followed by insertion in the corresponding apoenzyme (Schwarz et al. 2009; Iobbi-Nivol and Leimkühler 2012). The precise form of Moco in the YcbX and YiiM proteins has not yet been established, although it is clear that in contrast to most other *E. coli* molybdoenzymes it is not present as a MGD dinucleotide (Kozmin and Schaaper 2007).

**Figure 7:**
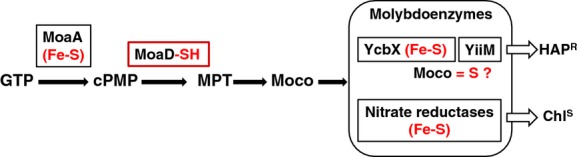
Possible roles of the IscS-TusA complex in Moco-dependent pathways. The possible stages where lack of IscS- and TusA-mediated sulfur mobilization could lead to sensitivity to HAP (loss of YcbX and YiiM activity) or to chlorate resistance (loss of nitrate reductase activity) are indicated in red font. The MoaD-SH box is outlined in red, as this stage is proposed to be the main critical step. See text for details.

The current observations on the HAP sensitivity and chlorate resistance of *tusA* and *iscS* mutants raise the question of the precise role of the TusA and IscS proteins in Moco-dependent pathways. In Figure 7, we have indicated several ways in which defects in sulfur metabolism could potentially interfere with Moco biosynthesis. Synthesis of Moco has a direct sulfur requirement as it contains two dithiolene sulfurs that serve to coordinate the Mo atom (Fig. 1C). In addition, its biosynthesis requires, among others, the MoaA protein, which is an iron–sulfur cluster protein (Schwarz et al. 2009; Iobbi-Nivol and Leimkühler 2012). The YcbX protein (for HAP resistance) as well as nitrate reductase enzymes (for chlorate sensitivity) are also iron–sulfur proteins, and their activity may be impaired in *iscS*/*tusA* strains for this reason. Finally, the hypothesis could be considered that Moco present in YcbX and YiiM contains a sulfur atom as an additional Mo-ligand, as demonstrated to be the case for enzymes of the xanthine oxidase family (Schwarz et al. 2009; Iobbi-Nivol and Leimkühler 2012).

With regard to the role in IscS, biochemical studies have shown this cysteine desulfurase to be the primary sulfur-donating factor in MPT synthesis (Zhang et al. 2010; Dahl et al. 2011), with no inferred role for the alternative CsdA and SufS cysteine desulfurases (Zhang et al. 2010; Dahl et al. 2011). Our results showing the HAP sensitivity and chlorate resistance of the *iscS* mutant are fully consistent with this possibility, including our observed lack of effect of the alternative *cdsA* or *sufS* cysteine desulfurase defects. In addition to this role, IscS is also the main (housekeeping) sulfur donor for the synthesis of iron–sulfur clusters in *E. coli* (Fontecave et al. 2008; Roche et al. 2013). In this function, IscS generally operates in conjunction with the IscU protein and a variety of additional factors like IscA, HscA, HscB, and Fdx, which are all expressed as part of the *iscRSUA-hscAB-fdx* operon (Zheng et al. 1998). Other Fe–S synthesizing systems, such as that encoded by the *sufABCDSE* operon or NfuA protein form alternative systems that may play a role in the repair of damaged clusters under stress conditions (Fontecave et al. 2008; Roche et al. 2013). Importantly, we showed that the *iscU* and *iscA* defects (or the alternative *sufA* or *nfuA* defects) did not produce any sensitivity to HAP (Fig. 6). As the IscU protein is required for Fe–S cluster biosynthesis by the IscS-IscU pathway, this leads us to two conclusions.

First, the role of IscS in MPT biosynthesis, which does not require IscU, is clearly distinct from its role in Fe–S cluster synthesis, which does require IscU. This observation is consistent with emerging models in which IscS is able to participate in several different processes but using different partner proteins (Shi et al. 2010; Maynard et al. 2012; Dahl et al. 2013). It is plausible to assume that, for MPT synthesis, IscS partners with TusA, to provide the sulfur atoms needed by the MoaD protein for MPT synthesis. This would be similar to the described IscS-TusA partnership that performs the thiolation of the wobble position of certain tRNAs yielding the modified tRNA base 5-methylaminomethyl-2-thiouridine (Ikeuchi et al. 2006). The physical IscS-TusA complex and its crystal structure have been described (Ikeuchi et al. 2006; Shi et al. 2010).

The second conclusion is that iron–sulfur cluster assembly per se does not appear to be a rate-limiting factor for the HAP-detoxification pathway, as the loss of the IscS-IscU pathway (as in the *iscU* mutant) does cause detectable HAP sensitivity. It appears that sufficient Fe–S centers must be present in the YcbX protein and MoaA protein (see Fig. 7). It is likely that some alternative IscU-independent pathway provides sufficient Fe–S clusters for these proteins to remain active. The one possible exception to this interpretation may be the observed chlorate resistance of the *iscU* mutant (Fig. 4). This aspect will be addressed further below.

The possibility (see Fig. 7) that an extra sulfur atom might be required as additional Mo-ligand in Moco in YcbX or YiiM, as found in the Xanthine Oxidase family (Schwarz et al. 2009; Iobbi-Nivol and Leimkühler 2012; Hille 2013), was also considered, as the precise nature of Moco in YcbX and YiiM is presently undetermined. Recent evidence indicating that the corresponding eukaryotic proteins, human mARC or green algal crARC, do not carry an additional Mo=S ligand on their Moco (Wahl et al. 2010; Chamizo-Ampudia et al. 2011; Rajapakshe et al. 2011). Also, our results show that *iscS* and *tusA* mutants are chlorate resistant (see Fig. 4), indicating a defect in nitrate reductase activity. All three *E. coli* nitrate reductase (NarGHI, NarZYV, and NapABCGH) are members of the DMSO Reductase family of molybdoenzymes and carry the Mo-bisMGD form of Moco, which does not contain the extra sulfur ligand (Hille 2013; Iobbi-Nivol and Leimkühler 2012). Thus, a parsimonious interpretation of these data would rule against a requirement of Moco in YcbX and YiiM for an extra sulfur ligand. Thus, the combined data argue in favor of the hypothesis that the observed HAP sensitivity of *iscS* and *tusA* mutants results from lack of MPT synthesis (Fig. 7).

Overall, the results with the chlorate-resistant phenotype of *iscS* and *tusA* paralleled those obtained with the HAP-sensitivity phenotype (Fig. 4). However, an interesting split-phenotype result was obtained with the *iscU* mutant. This mutant proved fully HAP resistant (Fig. 6), indicating sufficient YcbX/YiiM activity, but it was chlorate resistant (Fig. 4), indicating some loss of nitrate reductase activity. However, the chlorate resistance of *iscU* appears, in fact, to be only partial, as judged from their significantly smaller colonies on the chlorate plates compared to, for instance, the fully resistant *moaE* mutant (Fig. 4). We suggest that this partial nitrate reductase deficiency results primarily from a reduction of the iron–sulfur clusters on nitrate reductase, although an indirect effect on Moco via its effect on the MoaA iron–sulfur cluster could also play a role. A split phenotype of this kind (partial chlorate resistance along with full HAP resistance) has also been reported for certain polar mutants of the *moa* operon (Kozmin and Schaaper 2013). It appears that a modest reduction in nitrate reductase activity can already give rise to a significant level of chlorate resistance, while a much more robust reduction in YcbX/YiiM activity is required to observe HAP sensitivity (Kozmin and Schaaper 2013).

Another interesting observation of the current study is that the IscS/TusA-dependent step could be circumvented by the addition of sodium sulfide or L-cysteine to the growth medium (Fig. 5). It is possible that the MoaD protein can directly utilize inorganic sulfide, at least to a sufficient extent, to assure adequate Moco levels. Support for this idea comes from in vitro biochemical experiments in which activation of MPT synthase could be achieved by incubation with sodium sulfide (Leimkühler and Rajagopalan 2001). As our results show the same effect by addition of L-cysteine, L-cysteine may be catabolized in vivo to generate sulfide. Indeed, in *E. coli* excess of L-cysteine is readily removed by L-cysteine desulfhydrase activities, of which tryptophanase (*tnaA* gene product) has been identified as a main activity (Snell 1975). We have investigated *iscS tnaA* and *tusA tnaA* double mutants for HAP sensitivity and observed that in those cases addition of cysteine had much reduced effects, in support of this hypothesis (data not shown). Likewise, an increase in the intracellular cysteine or sulfide concentration in the absence of any additions to the media was also achieved (data not shown) by constitutive expression of the CysB regulon, which normally controls the cysteine biosynthesis pathway (Kredich 1996).

The recent study by Dahl et al. (2013) has also described a role for TusA in the thiolation reaction that produces MPT. However, somewhat different conclusions were reached. Biochemical analyses of MPT content revealed a low MPT content in *tusA* cells growing aerobically, but only an about 50% reduction for MPT in anaerobically growing *tusA* cells. Hence, while it was acknowledged that in aerobic cells TusA may be a preferred IscS partner for the cPMP thiolation reaction, TusA should not be regarded as an obligate IscS partner for this reaction in view of the anaerobic results. Instead, alternative sulfur carriers such as YnjE might play a role (Dahl et al. 2013). It was also observed that *tusA* cells suffer from altered gene expression patterns, possibly resulting from altered cellular Fe–S status (Maynard et al. 2012), and it was suggested that the effects of the *tusA* deficiency might result indirectly from these altered patterns (Dahl et al. 2013). Pleiotropic effects of a *tusA* deletion have also been reported in other studies (Yamashino et al. 1998; Ishii et al. 2000; Nakayashiki et al. 2013). It thus appears that the precise role of TusA in MPT synthesis requires further investigation. Our experiments measuring the effects of IscS and TusA on HAP resistance were performed under aerobic conditions and, hence, our results are consistent with the strong reduction in MPT observed under these conditions. On the other hand, chlorate-resistant experiments are performed under anaerobic conditions. Hence, our observation that *tusA* strains are chlorate resistant (Fig. 4) must indicate that even under these anaerobic conditions other hypothetical sulfur-carrier proteins cannot readily substitute for TusA. Experiments with a Δ*ynjE* strain (results not shown) revealed normal HAP resistance (aerobic condition) as well as normal chlorate resistance (anaerobic condition), suggesting that the role of YnjE protein as alternative sulfur carrier may be limited.

Our genetic studies with the *N*-hydroxylated base analogs have provided a sensitive window into several important biological phenomena. Detoxification of hazardous agents, either produced endogenously or encountered in the environment, is an important cellular activity. The severe sensitivity of Moco-deficient strains has enabled the discovery of the YcbX and YiiM enzymes that represent a novel class of activities. The question of the physiological substrate of these enzymes is still an open question, and deserves further investigation, especially in light of the discoveries of corresponding mammalian enzymes (mARC) that have a mitochondrial membrane localization (Wahl et al. 2010; Klein et al. 2012). Furthermore, the precise metabolism of endogenous or foreign toxicants is an issue that can be further explored using this experimental system. Additional results from our laboratory have revealed that exposures to HAP have profound consequences for cellular DNA/RNA and nucleotide metabolism, including the involvement of cellular response systems, like the SOS system (Foster 2007). Most importantly, the results have allowed new insights into the role and metabolism of Moco in the cell and have defined an important, novel role of the TusA-IscS complex, as shown in the present study.
